# Supplementary material for: Are carbon emissions trading and green financial instruments synergistic? -Comprehensive quantitative research based on content analysis
Source: PLoS One. 2024 Mar 7;19(3):e0298601. doi: 10.1371/journal.pone.0298601 (PMC10919601; doi:10.1371/journal.pone.0298601)
Supplement: S1 Data — (ZIP) [file pone.0298601.s001.zip › Data/地级市Z_F工作报告.zip!/║■▒▒/╧σ╤⌠╩╨/└┤╫╘╣1⁄2╓┌║┼í╛┬φ┐╦╩2╛▌═°í┐_2010.docx]

各位代表：

　　现在我代表市人民政府向大会作工作报告，请予审议，并请政协委员和其他列席人员提出意见。

　　一、关于2009年的政府工作

　　2009年，面对国际金融危机的巨大冲击，市政府紧紧依靠省委、省政府、市委的正确领导和市人大、市政协的监督支持，团结和带领全市人民，以科学发展观统揽全局，围绕实现地区生产总值四年翻番的宏伟目标，按照市十五届人大二次会议工作部署，解放思想，迎难而上，全力以赴保增长、保民生、保稳定，全市经济社会呈现又好又快发展新局面。

　　(一)应对危机措施有力，经济增长超出预期

　　针对金融危机背景下经济运行中的突出矛盾和问题，我市全面落实中央促进经济增长的各项政策，果断采取有效措施，帮助市场主体抗御危机冲击，全市经济经受住了严峻考验，迅速企稳回升，步入上升通道。初步核算，2009年全市地区生产总值1201亿元，同比增长15%。全社会固定资产投资增幅、工业投资增幅、外商直接投资增幅、规模以上工业增加值增幅、新增规模以上工业企业数、农业增加值增幅、粮食增产量等七项经济指标均居全省第一位。

　　工业经济强势回升。2009年，全市工业平开高走、逐月提速，完成规模以上工业产值1528亿元，同比增长40.2%；规模以上工业增加值461.5亿元，同比增长27.6%。汽车产业在东风板块的带动下实现31.4%的强劲增长，完成产值703.8亿元；食品、装备制造和纺织业产值分别达到272.59亿元、202.57亿元和 146.16亿元，分别增长72.4%、28.5%和35%；化工医药和电子信息行业成为新的百亿产业，全市百亿产业达到6个。规模以上工业企业净增305家，达到1306家；产销过20亿元的企业发展到11家。“奥星”牌食用植物油等28个产品被评为湖北名牌产品，华中药业的“华意”等39个商标成为“湖北省著名商标”。

　　现代农业发展加快。农产品加工业迈上新台阶，产值达到460亿元，同比增长58.7%；产销过亿元的企业达到56家，其中6家企业突破10亿元；老河口农副产品加工园成为全省首个农业产业化示范园区。农业综合生产能力增强，新建高产农田27.7万亩，恢复改善灌溉农田50万亩，新建国家和省级农业标准化示范区8个、农业科技创新基地11个、万亩粮棉油优质高产示范片49个、规模养殖场62个、养殖小区221个。粮食总产量达到433.25万吨，比上年增产20.13万吨。

　　消费市场繁荣活跃。家电和汽车下乡等一系列促进消费政策全面落实，城乡消费持续升温，社会消费品零售总额500.5亿元，同比增长21.1%。市区万达广场、东风合运物流中心等一批现代商贸物流项目正在抓紧建设。出口形势逐步好转，完成出口额3.29亿美元，降幅由2月份的34.9%收窄至12.2%。旅游活市战略正式确立，完成旅游项目投资3.3亿元，是上年的1.1倍；谷城县被评为“湖北旅游强县”，尧治河等8个村成为“湖北旅游名村”；全市接待游客997.2万人次，同比增长21.17%。

　　区域经济强劲增长。14个开发区完成固定资产投资293.7亿元，同比增长81.6%。深圳工业园一期基础设施建成并投入使用，襄城经济开发区、航空航天工业园、三环襄轴工业园、力天鞋业工业园、光彩工业园等一批特色园区建设快速推进。完成规模以上工业产值1286.2亿元，同比增长42.6%；其中高新区规模以上工业产值652亿元，同比增长26.5%。县域经济快速增长，省考核的7个县市区地区生产总值、城镇以上固定资产投资、规模以上工业增加值和地方一般预算收入增幅分别达到16.2%、73.6%、51.4%和28.3%；占全市的比重分别达到56.6%、57.4%、43.1%和46%，分别比上年提高3.1、6.7、7.7和1.9个百分点；襄阳、谷城、枣阳、宜城、老河口等5个县市区规模以上工业产值突破百亿元大关。

　　(二)投资规模明显扩大，项目支撑进一步加强

　　项目建设卓有成效。完成全社会固定资产投资574.8亿元，同比增长53.8%。新开工项目1822个，同比增加574个；120个投资过亿元重点项目完成投资222亿元，33个竣工投产。完成工业投资305.8亿元，同比增长67.4%，东风A08轻型客车、龙蟒集团磷酸盐等59个投资过亿元的工业项目建设快速推进，际华3542家纺、葛洲坝水泥等25个项目建成投产，汉江崔家营航电枢纽2台机组并网发电，工业发展后劲明显增强。

　　招商引资实现新突破。全市新增招商注册项目607个，总投资689亿元；其中亿元以上项目98个，总投资538亿元，中航集团、新兴铸管集团、三环集团、同济堂医药公司等10个企业项目投资均在20亿元以上。全市在建招商项目608个，总投资681亿元；投产项目340个，完成投资138亿元。国有企业改革重组取得新成效，三环集团重组襄轴、兵装集团华中药业重组湖北制药进展顺利。对外交往和利用外资取得积极成效，全市新批外商投资企业25家，直接利用外资2.5亿美元，同比增长48.1%。与此同时，争取国家和省投资项目666个，无偿资金13.42亿元。全民创业投资形势喜人，新增个体私营经济组织8742个，注册资本18.2亿元。

　　(三)经济质量不断提高，发展方式转变加快

　　经济效益不断改善。规模以上工业实现主营业务收入1278.6亿元，同比增长35.3%；实现利税116.8亿元，其中利润62.3亿元，同比分别增长36.6%和60%。亏损企业亏损额同比下降58.1%。在政策性减税额度较大的情况下，全市财政总收入仍完成95.13亿元，同比增长15.4%；地方一般预算收入达到37.02亿元，同比增长23.2%；税收占一般预算收入的比重达到72.7%。财力增加有力保障了工资发放、民生和社会建设等公共支出，实现了财政收支平衡。

　　新兴产业蓬勃兴起。以东风天翼纯电动客车、高新青山和宇清电动汽车为主的新能源汽车产业扎实起步，节能与新能源汽车公交示范线路获得省政府批准运营；一批航空航天、节能环保和生物医药产业项目开工建设，可望形成新的产业支撑。 与此同时，企业和区域创新能力进一步提升。相继实施30个产学研合作项目、80个国家和省科技计划项目，13家企业列入全省科技型中小企业成长路线图计划，新增1个国家级企业技术中心、9个省级企业技术中心、工程技术研究中心和检验检测中心，新增36家高新技术企业、1个国家级和14个省级创新型企业，谷城县和宜城市分别成为国家级和省级可持续发展实验区，我市被评为全国科技进步示范市。完成高新技术产业增加值 184.7亿元，同比增长33.8%。

　　节能环保措施加强。关闭5家小造纸厂、4家小印染厂和3家小水泥厂，20个节能技改项目获得国家财政支持，我市被列为全国首批可再生能源建筑应用示范城市；建成1个市区污水处理厂和3个县级城市污水处理厂，启动了市区生活垃圾集中处理焚烧发电工程。预计单位生产总值能耗下降4.8%，化学需氧量下降2.53%，二氧化硫减排提前超额完成“十一五”规划目标，汉江干流水质保持在Ⅰ-Ⅲ类之间。在完成林权制度改革的同时，加强生态建设，人工造林 22.84万亩，新增自然保护区面积约6万公顷。

　　(四)发展平台明显优化，发展环境持续改善

　　城市功能进一步提升。市区内环线西段、北段建成通车，汉江三桥正抓紧建设，东外环线正式开工，学院路、机场路等一批城市路网改造和21个防汛排涝设施工程竣工，鱼梁洲河道疏浚和护堤建设完工并新增土地7600亩。新开通市区公交线路3条51公里。县级城市建设加快，南漳县城凤凰大道建设、老河口市滨江大道改造、保康县城综合改造等一批重点项目进展顺利。区域交通枢纽功能进一步增强，改造国(省)道干线和县乡公路200多公里，麻竹高速襄樊段等 6条高速公路建设项目和207国道市区段等11条国(省)道干线改扩建项目列入国家和省规划；襄樊至武汉高速铁路动车及航线相继开通，至北京、上海、广州、深圳航班进一步加密，襄樊机场周航班量达到32个，年旅客吞吐量达到13.7万人次。

　　要素支撑力度加强。争取金融机构贷款授信额598亿元，金融机构存贷款余额分别突破千亿元和500亿元大关，贷款比年初净增144.7亿元，是上年的2.5倍，有力支持了经济社会发展。引进金融机构实现新突破，中信银行襄樊分行正式开业，光大银行、招商银行、华夏银行和民生银行筹建襄樊分行的前期准备工作顺利进行。市建投公司、商业银行以及14家中小企业担保机构实力明显增强。企业上市工作取得重大突破，回天胶业和台基股份股票成功登陆创业板。与此同时，千方百计保障土地、电力等重要生产要素供给，申报建设用地计划2.47万亩，比上年增加1.1万亩；社会用电量和工业用电量同比分别增长6.7%和4.9%。“隆中人才计划”深入实施，成功引进了一批高层次人才。

　　“阳光新政”取得新成效。在深化工业项目审批改革的同时，全面推进建设类、便民类和非工业类事项审批改革，审批程序得到简化，审批时限明显缩短，行政规费大幅减免，市行政服务中心受理行政审批事项承诺期内办结率达100%。深入开展“群众评科长”、“千家企业评机关”和“能力建设年”活动，严肃查处214起行政不作为、乱作为案件，政风、行风进一步好转。同时，按照中央和省部署，全面启动了新一轮政府机构改革。积极争取市厅合作，与35个省直厅(局)签署了合作协议，为我市发展赢得了更多的支持。

　　(五)民生建设继续加强，社会事业发展加快

　　市政府年初向全市人民承诺的十件实事全部完成。

　　就业工作力度加大。培训城乡劳力 10.4万人；组织外地企业和本地新建、扩产企业提供就业岗位3万个，开发公益性岗位3267个，新增自主创业主体7100个、回归创业企业902个，就业渠道进一步拓宽。全市城镇新增就业人员 9.77万人，城镇登记失业率控制在4.18%以内；新增转移农村劳力10.13万人，14.56万名返乡农民工实现再就业。

　　社会保障水平进一步提高。社会保险覆盖面扩大，城镇居民医保和新型农村合作医疗实现愿保尽保，村主职干部养老保险、被征地农民和残疾人社会保险制度初步建立，新型农村养老保险试点工作扎实推进。社会救助力度加大，30万城乡困难居民享受最低生活保障待遇。住房保障加强，住房公积金归集和贷款规模进一步扩大；改造农村危房1649户，新建城市廉租房3881套，改造棚户区3.52万平方米，向2.3万余户困难家庭发放了住房租赁补贴。农民人均纯收入和城镇居民人均可支配收入分别达到5440元和13409元，同比分别增长11.5%和9.1%。居民消费价格下降1.3%。南水北调移民安置工作全面启动，向大中型水库原迁移民发放直补6943万元。

　　公共事业实现新发展。教育水平进一步提高，初中毕业生升学比例达到 88%，高中阶段教育基本普及，高考本科上线率达到 52%以上。义务教育均衡发展取得新成效，农村寄宿制学校学生住宿费全部免除；进城务工人员子女义务教育得到确保；市区24所薄弱学校设施改造基本完成；筹资1.7亿元，资助困难学生20余万人次。医药卫生体制改革稳步推进，54个基层卫生服务项目建成并投入使用，村卫生室和城镇社区卫生服务机构覆盖率分别达到 98%和100%；甲型H1N1流感、手足口病等重大疫病防控扎实有效。人口与计划生育政策深入落实，出生政策符合率达到92.3%，出生人口性别比趋向正常。

　　应急管理和安全生产监管加强。政府应急管理体系和机制进一步完善，及时妥善处理各类突发事件160余起。气象、地震、地质等自然灾害防灾减灾工作进一步加强。消防、非煤矿山、道路交通、特种设备、危险化学品和烟花爆竹安全整治工作持续深入，食品、药品安全监管力度加大，我市安全生产事故指标控制在省政府下达的目标以内，有力维护了人民群众的生命和健康权益。

　　新农村建设扎实推进。全市形成了片线面结合、市县镇联动的新农村建设新格局，9个省级新农村建设试点镇工作全面启动， 152个村达到示范村庄整治标准，襄南新农村示范区建设经验得到了省委、省政府充分肯定。保康脱贫奔小康试点县建设取得积极进展。农村公共设施建设加快，解决了52.47万人的饮水不安全问题，新增农村沼气用户4.2万个，新建通村水泥(油)路2500多公里，村通客车率达97.5%，人居环境进一步改善。

　　(六)民主法制建设加强，精神文明建设取得新成果

　　法治襄樊建设进一步深化。市政府始终坚持自觉接受市人大及其常委会的法律监督和市政协的民主监督，认真办理人大代表议案、建议和政协委员提案；密切联系工会、共青团、妇联等人民团体，广泛听取民主党派、工商联、无党派人士的意见建议，促进了民主决策和科学决策。加强法制宣传教育，严格实施依法行政目标考核，着力化解行政争议，规范行政处罚机关处罚自由裁量权，各级依法行政水平进一步提高。

　　信访维稳工作扎实有效。认真处理群众来信来访，为困难群众提供法律援助，强力整治社会治安，严厉打击各类犯罪，有力维护了社会稳定，我市被评为“全国社会治安综合治理优秀市”，信访维稳工作的做法得到中央领导充分肯定，并在全省推广。

　　城乡文明程度进一步提高。我市被评为“湖北省文明城市”、“全国创建文明城市工作先进城市”和“全国民族团结进步先进市”。襄樊供电公司等3个单位获得“全国文明单位”称号，市审计局等4个单位成为“全国精神文明建设先进单位”，五山镇等5个镇、村成为“全国文明村镇”，樊城区成为“全国和谐社区建设示范城区”。文化服务体系建设加强，建成了 10个乡镇综合文化站、550个农家书屋和13个城市社区流动图书站，新建了90个村和20个城市社区的健身设施，完成了1500个20户以上自然村广播电视“村村通”工程；以庆祝新中国成立60周年为主题的文化活动丰富多彩，人民群众精神文化生活进一步改善。“双拥”工作进一步加强，驻樊解放军、武警官兵和民兵预备役人员为地方经济社会发展做出了积极贡献。

　　与此同时，国有资产监管、统计、人防、老龄以及其它各项政府工作都取得了新的成绩。

　　各位代表，过去一年，全市经济社会发展能够在遭遇国际金融危机巨大冲击情况下保持良好态势，主要是得益于中央和省一系列保增长措施的全面落实，得益于我市生产总值四年翻番战略的确立和实施，得益于全市风清气正、团结干事的政治环境，得益于全市人民同心同德，奋力拼搏。我代表市政府，向全市人民，向各位代表、各位委员，向各民主党派、工商联、人民团体和驻市单位，向驻樊人民解放军、武警官兵，向所有关心支持襄樊发展的同志们、朋友们，表示衷心的感谢，并致以崇高的敬意！

　　尽管过去一年的政府工作取得了一定成绩，但我们也清醒地看到，困扰我市发展的矛盾和问题仍然较多，主要是：全球金融危机的不利影响尚未完全消除，部分行业、部分企业仍未完全走出困境；支撑发展的大项目不多，招商引资的质量和水平有待提高；经济结构不优，新兴产业发展滞后，产业竞争力有待提升；农业基础设施建设和农产品加工业发展仍然不够，新农村建设水平与广大农村群众的生产生活需求相比仍有较大差距；群众反映强烈的一些民生问题尚未完全解决；制约发展的观念和体制机制障碍尚未有效破除，部分政府部门和公务人员思想解放不够、开放意识不强、行政服务不优的问题还比较突出。对于这些矛盾和问题，我们将采取积极有效措施，认真加以解决。

　　二、关于2010年政府工作目标和任务

　　2010年既是“十一五”规划结账年，也是实施生产总值四年翻番规划的关键年，做好今年的工作，对于实现跨越式发展目标至关重要。当前，我市经济社会发展条件十分有利。从宏观环境看：世界经济开始复苏，我国经济全面复苏，尤其是汽车、纺织、电力、化工医药、建材冶金等行业日趋回暖，为我市相关产业发展提供了有力的需求支撑。中央决定继续实施积极的财政政策和适度宽松的货币政策，加大财政对民生领域、节能环保、中小企业、居民消费和欠发达地区的支持，加大信贷对经济社会薄弱环节、就业、战略性新兴产业、产业转移、中小企业发展和重点项目建设的支持，这些政策措施蕴含着重大发展机遇。中央采取有力措施扩大内需，尤其是继续实施汽车下乡和以旧换新政策，有利于我市汽车工业加速发展；增加涉农补贴规模，提高主要粮食品种最低收购价格水平，加大农业基础设施投入，有利于我市加快由农业大市向农业强市转变；国家促进中部崛起措施加强，湖北实施“两圈一带”和汉江流域综合开发战略，为我市培植新的经济增长点带来了重大契机。从我市发展态势看：近几年实施的“三个百亿工程”成效显著，一大批项目竣工投产，经济步入加速发展的上升通道，跨越式发展支撑有力；同时，通过深入学习实践科学发展观，一心一意谋发展、同心同德抓发展，已成为全市上下的广泛共识和一致行动。这些都为做好今年政府工作，实现更好更快发展，提供了十分有利的条件。

　　今年政府工作的总体思路是：以党的十七大、十七届四中全会精神为指导，按照十一届市委十次全会总体部署，紧紧围绕生产总值四年翻番宏伟目标，深入落实科学发展观，大力实施“双千双百工程”，强化招商和项目建设，加快扩张经济规模，提升壮大优势产业，积极发展新兴产业，高度重视节能环保，加强民生和社会建设，努力转变发展方式，提高发展质量和效益，全面完成“十一五”规划确定的目标任务，为建设更加开放繁荣、更加文明和谐的现代化大城市，奠定坚实的基础。

　　根据上述思路，按照四年翻番的要求，今年全市经济社会发展的预期目标是：地区生产总值增长14%，规模以上工业增加值增长20%，全社会固定资产投资增长31%，社会消费品零售总额增长20%，直接利用外资和出口额分别增长15%和12%以上，地方一般预算收入增长15%，城镇居民人均可支配收入和农民人均纯收入分别增长9%和7%；约束性目标是：城镇登记失业率控制在4.5%以内，人口出生政策符合率达到91%以上，单位生产总值能耗下降4%，化学需氧量和二氧化硫排放量分别控制在5.71万吨和7.83万吨以内。为实现上述目标，我们将重点做好六个方面的工作：

　　(一)着力扩大投资规模，强力拉动跨越式发展

　　坚持以大投资拉动大发展的举措不动摇，确保完成全社会固定资产投资 750亿元，实现两年翻一番的目标。

　　突出重点项目建设，加快扩大产业投资。加快推进133个投资过亿元的重点项目建设，突出抓好34个投资过10亿元的重大项目建设，力争当年完成亿元以上项目投资 320亿元。大力推进东风A08轻型客车等66个先进制造业项目建设，力争当年投资100亿元；大力推进万达广场等11个现代服务业项目建设，力争当年投资34亿元；大力推进开发区基础设施等43个基础设施项目建设，力争当年投资162亿元；大力推进农村安全饮水等13个农业农村、生态环境和社会发展项目建设，力争当年投资24亿元以上。积极争取国家和省支持，加快实施汉江综合开发，为投资大扩张、产业大发展注入强劲动力。在依法严格保护耕地、促进集约节约用地的同时，加强土地储备和供应，确保重点项目建设用地需求。

　　强化招商引资举措，着力引进重大投资。积极扩大对外交流，巩固和发展与国内外发达城市和商会的友好合作关系，着力培育和扩大招商资源。优化招商政策，形成比较优势，增强招商竞争力。整合市县招商力量，抽调一批优秀干部，培训和选聘一批专业人才，组建高素质、专业化的招商队伍，提高招商工作水平。完善市场主体招商激励机制，鼓励国内外资本开发工业园区，自主开展招商引资；支持龙头企业利用配套市场，引进上下游企业，延伸产业链，做大产业集群；抓住国企重组加速的机遇，争取中央和省属企业加大在我市的投资力度；抓住国家促进结构调整的机遇，争取行业龙头企业换代升级项目布局襄樊。力争每个县市区引进1个投资过10亿元、2个投资过5亿元的大项目，全市新增注册招商项目投资600亿元以上。

　　加强资本市场建设，争取扩大金融投资。进一步优化金融信用环境。支持金融机构在优化结构、防范风险的同时，扩大信贷规模，力争贷款余额达到700亿元左右。争取光大银行、招商银行、华夏银行和民生银行襄樊分行早日开业运营，鼓励有实力的企业兴办小额贷款公司和村镇银行，支持农村信用社加快改组为农村合作银行，引导银行业多元化发展。争取上级金融机构选派干部到我市挂职，促进银政企合作。鼓励金融机构服务中小企业，提升担保机构担保能力，努力解决中小企业融资难；积极争取国家创业投资基金参股，促进我市创投公司做大做强，大力引进新的创业资本，支持中小企业发展。加快推进资产证券化，争取证监会受理我市 2-3家企业上市申请，在进军新三板市场上取得进展。支持骨干企业利用股权融资、债券融资，实现跨越式发展。

　　(二)着力实施“双千双百工程”，加速工业扩张升级

　　把实施“双千双百工程”作为产业发展的总抓手，加快培育千亿级产业、百亿级企业、千亿级高新区和百亿级开发区。

　　突破性发展优势产业，加速扩张工业规模。举全市之力推进汽车产业加速迈向千亿级目标，力争今年产值突破900亿元。大力支持东风汽车股份公司实施三年内实现产销 30万辆整车和30万台发动机的发展规划，支持风神襄樊公司 15万辆中高档轿车扩能项目建设，支持神龙公司襄樊工厂尽快形成67万台发动机和45万台变速箱的产销能力，支持东风德纳车桥公司实施产销过百亿元发展规划；加快建设东风公司、江山重工和湖北福田专用车项目，尽快形成产业规模；促进零部件企业积极跟进龙头企业，加快发展配套产业集群。着眼于培育千亿级产业，突破性发展食品工业，重点抓好襄樊烟厂、蓝带啤酒、东汇食品等11个投资过亿元的项目建设，力争食品工业产值突破400亿元。加快发展轻纺工业，重点推进际华3542汉麻面料开发、力天制鞋等12个投资过亿元的骨干产业项目建设。壮大装备制造和化工医药产业，支持新兴重工、金鹰轨道、新火炬、三环襄轴等装备制造龙头企业扩大产能；大力推进同济堂药业、华中药业、湖北制药、湖北天药和隆中药业的生物医药项目建设，促进龙蟒集团、兴发集团、楚磷公司、泽东公司磷化工项目尽快投产。力争建材冶金行业跨入百亿产业行列，重点抓好美亚达彩涂铝板、中部三农环保秸秆板等12个投资过亿元的项目建设。大力实施企业成长工程，制定并实施产销过 10亿元、50亿元和百亿元企业培育计划，促进中小企业加速成长，新增规模工业企业200户。实施质量兴市战略，力争再创一批国内名牌产品和著名商标。

　　突破性发展新兴产业，加速推进产业升级。加快建设东风旅行车、高新青山、襄樊宇清、天舟科技、江苏新日、骆驼蓄电池等骨干企业的电动车产业项目，着力打造全国重要的新能源汽车产业基地。加快建设大唐电力新集电站、雅口电站、杨家峡电站、凯迪电力生物质能源等新能源项目，支持安能生物质热电公司发展，力争在发展LED照明、风电装备和光伏产业上实现突破，着力打造全省重要的新能源产业基地。加快建设中航集团、航天科技集团、5713工厂、航奥伺服科技公司等企业的重点项目，突破性发展航空航天产业，着力打造中部地区新兴的航空航天产业基地。加快建设一批节能环保产业项目，尽快形成产业规模，着力打造全省重要的节能环保产业基地。支持以大力、万洲、追日、国网绝缘子为龙头的电力设备行业抢抓国家智能电网建设机遇，着力打造国内一流的智能电网产业基地。以创建国家创新型城市为契机，加快建立以市场为导向、企业为主体、产学研结合的区域创新体系。建立健全以创业孵化、研究开发、公共技术服务、投融资、专利交易和产品质量检验检测为重点的创新服务平台，着力扶持和培育创新型企业，促进产业联盟建设，壮大新兴产业发展主体。支持军工企业发挥人才、技术和资本优势，积极发展战略性新兴产业。大力实施“隆中人才计划”，支持企业建立院士和博士后工作站、博士后产业基地，加强高新区专家院士服务中心建设，引进一批高层次创新创业团队和专业技术人才，强化新兴产业发展的人才支撑。

　　突破性发展开发区，加速推进产业集聚。以建设千亿级高新区为目标，加快建设襄轴工业园、东湖高新工业园和生物医药产业园，规划建设新能源汽车产业园、特种汽车产业园和乘用车零部件产业园；配套建设汽车工业园综合服务区、武商大型综合超市和车城湖公园，实施园中村改造，拓展发展空间，提升高新区综合服务和产业集聚功能；力争高新区主要经济指标增幅高于全国国家级高新区平均水平，规模以上工业产值突破850亿元。采取有效政策措施，集中各种经济要素，加快推进省级开发区建设和发展。启动深圳工业园基础设施二期工程建设，加大项目引进和建设力度，力争新增注册项目40个以上，其中投资过亿元的项目 20个左右，投产项目20个以上。完成航空航天工业园和余家湖工业园一期基础设施建设，力争在引进和建设航空航天产业项目上实现新突破，余家湖工业园基础设施建成区实现项目满园。支持县市区开发区加快发展，集中连片建设10平方公里左右的工业新城，促进1-2个开发区规模以上工业产值突破百亿元；力争各县市区在全省县域经济考核中全部进位，1-2个县市区进入全省县域经济20强。

　　(三)着力发展现代农业，加快推进新农村建设

　　加快发展农产品加工业。加速推进农产品加工业向千亿级产业迈进，力争今年产值突破 600亿元。以培育全省乃至全国行业龙头为目标，加快引进品牌知名度高、市场竞争力强的龙头企业；支持粮油、白酒、森工和畜禽加工等行业重点企业扩大产能，通过相互持股、控股等方式联合重组，共创品牌，做大做强；大力支持县市区农产品加工园建设，力争我市农产品加工业发展水平跃居全省前列。创新现代农业发展机制，大力推行龙头企业+基地+农户的产业化经营模式，大力发展农民专业合作社；深化集体林权制度配套改革；加快建立乡镇农村土地经营权流转服务中心，促进适度规模经营。

　　加强农业综合生产能力建设。抓好 145个涉农项目建设，增强农业发展后劲，粮食产量稳定在400万吨以上。以加工业为依托，扩大优质粮油、森工原料和畜禽养殖规模化、标准化生产基地，加快形成“一县一品”格局。扎实推进科技兴农，集成技术、集合项目，创建一批优质、高产、安全、环保农业生产示范区，力争新增绿色、有机、无公害农产品标识40个，无公害农产品基地认证面积扩大到500万亩。加强农田水利设施建设，加快推进“一江两河”堤防标准化整治，完成99座病险水库除险加固工程，全面疏浚渠系，清淤当家堰塘，恢复水毁工程，提高农业抗御自然灾害的能力。

　　加大统筹城乡发展力度。把推进城镇化作为统筹城乡发展的重要举措。放宽城镇户籍限制，鼓励有稳定职业和收入的农民工及其子女进城落户；加强城镇规划和管理，推进重点城镇基础设施建设，提高综合承载能力。坚持片、线、面结合，整体推进新农村建设。全面完成乡村建设规划。扎实办好9个省级新农村建设试点，加快推进襄南示范区扩面连片和县市区示范乡镇建设，着力整治汉江沿岸和铁路、国(省)道沿线村庄环境。把扶贫开发与新农村建设结合起来，抓好 116个村的整村推进工作，支持保康县做好脱贫致富奔小康试点工作。加快推进农村交通、饮水、沼气等基础设施建设，改善农村发展条件。积极探索利用社会资本推进新农村和小城镇建设的新机制。全面落实扶农、惠农、强农政策，千方百计促进农民收入持续增长。

　　(四)着力实施“旅游活市”战略，加快发展现代服务业

　　加快发展生态文化旅游业。着眼于打造国内外知名旅游胜地，高起点规划和建设核心景区。强化市区旅游龙头作用，强力推进古隆中景区扩容开发，启动岘山城市森林公园一期工程，加快鱼梁洲、鹿门寺和凤凰山水温泉度假村开发步伐；同时，支持县市特色景区建设，加快推进谷城薤山南河、保康九路寨和南漳古山寨旅游资源深度开发。在科学规划的基础上，大力发展文化产业，促进文化与旅游有机融合、互动发展，积极引资建设米芾文化产业园，力争在打造精品文化景观上实现突破；创新旅游发展体制机制，完成经营性景区企业化改造，实现景区运营市场化；创办旅游发展投资公司，加大旅游招商力度，拓宽旅游投融资渠道。完善旅游配套设施，加快建设旅游公路；规划建设旅游购物街区、特色饮食服务街区和文化娱乐休闲街区，促进酒店业提高旅游服务功能和水平。强化旅游营销，高水平策划、大手笔宣传襄樊旅游形象；在支持本地旅行社做大做强的同时，积极引进国内外知名旅行社，促进景区与旅行社缔结战略联盟，联合开发旅游市场。

　　加快发展现代服务业。着力引进和培植大型现代流通企业，加快建设万达广场、中心商业区等一批重点商业设施，规划建设特色商业街区和商业网点，启动建设市区现代小商品市场，提升市区商贸服务业发展水平。全面落实家电下乡、汽车下乡和以旧换新政策，继续推进万村千乡市场工程和新网工程，办好农村综合服务中心建设试点，新建和改造一批农家店、农资店，建设一批县级商品配送中心，促进农村消费升级。加快建设襄樊粮油仓储及农副产品现代物流中心，改造一批农产品批发市场和农贸市场，鼓励大型连锁超市和农产品流通企业在生产基地建设冷链物流系统、检测系统和配送中心，促进农超对接，搞活农产品流通。大力发展工业物流，重点抓好高新区物流园和汽车物流产业园项目建设。完善口岸、商检功能，积极申报设立航空口岸和保税物流园区，强化出口扶持措施，努力扩大对外贸易。积极探索发展服务外包、工程咨询、信息服务、市场中介、创意产业等现代服务业。

　　(五)着力完善城市功能，加快建设适宜人居、适宜企居的现代化大城市

　　完善城市重大设施。抓好34个城市路网、供水供气、防洪排涝、污水管网、环卫设施和城市维护等基础设施工程，加快建设内环线，抓紧建设汉江三桥、五桥和唐白河大桥，加快实施邓城大道、长虹路、襄隆路综合整治改造和207国道、316国道改线工程，开工建设通达县市的五条高速路。把开发区作为新的城市空间规划建设，优化城市功能布局。推进航空、铁路、高速公路、商检、口岸设施和信息化建设，促进服务协同、功能融合，增强综合服务功能。发挥市场配置资源的优势，多渠道筹集建设资金，加快城市建设步伐。

　　培育城市生态景观。实施城市森林生态工程，抓好汉十公路沿线襄樊段生态景观建设和市区外环线绿化试点。规划建设岘山城市森林公园。以“三口三线一路”为重点，打造城市生态屏障、绿色长廊和景观大道。启动市区汉江岸线景观带规划和建设，完成汉江北岸二桥至三桥段开发规划，实施一桥至鱼梁洲大桥段整体开发，打造滨江城市新景观。

　　加强城市规划和管理。建立城市重大建设规划方案竞选机制，面向国内外一流设计团队招标选择设计方案，提高城市规划设计水平。启动市行政中心建设规划，做好东津新区规划研究。严格执行城市规划，扩大城中村改造试点范围，坚决制止违法建设。实行“市区共管、以区为主”的城市管理体制，明确市区两级城市管理事权，强化城区、开发区管理城市的主体责任，加大城市管理力度；大力推进全国文明城市、国家环保模范城市和国家卫生城市创建工作，促进城市管理水平进一步提高。支持县市区加强城市规划、建设和管理，增强区域发展承载功能。

　　(六)着力加强民生建设，加快构建和谐襄樊

　　加强就业和社会保障工作。千方百计扩大就业。坚持以创业带动就业，综合运用各级政府鼓励创业、促进就业、支持非公有制经济发展的政策措施，促进县市区创业园区和创业孵化中心建设，大力支持全民创业，力争全年新增创业主体5000户以上；建设家政服务平台，扶持家政服务业发展，拓宽就业渠道。加大人力资源市场建设和就业岗位开发力度，重点做好城镇困难居民、失地农民、农民工、大中专院校毕业生和退伍军人就业技能培训和安置工作。加快建设覆盖城乡的社会保障体系。做好养老保险省级统筹工作；办好新型农村社会养老保险试点。努力增加中低收入居民的收入，认真落实国家提高城乡低保标准、企业退休人员养老金和优抚对象待遇的政策，提高城市社区工作者和环卫工人工资待遇，全面实施事业单位绩效工资制度。进一步提高城镇居民医疗保险、新型农村合作医疗的参保率和住院报销比例。加大住房公积金归集力度，加快建设廉租房，改造棚户区，增加保障性住房供应；认真落实国家房地产政策，促进房地产市场健康发展，构建合理的住房供应体系。大力发展社会化养老、慈善和残疾人事业，兴建市老年公寓，新建、扩建一批农村福利院。切实做好 2.1万名南水北调工程移民接收安置工作。

　　大力发展社会事业。优先发展教育事业。实施义务教育均衡发展行动计划，抓好校舍安全工程建设，进一步改善办学条件。确保进城务工农民工子女接受义务教育。加强高中阶段教育，力争90%以上的初中毕业生升入高中阶段学校。加快发展高等教育，支持襄樊学院争取开办农学院和医学院，提高办学水平；支持襄樊职业技术学院主校区建设，增强办学实力；积极申办汽车职业技术学院，力争在引进国内外资本兴办地方高校上实现突破；支持襄樊技师学院创建全国示范技师学院。以建立覆盖城乡居民的基本医疗卫生制度为目标，加快推进医药卫生体制改革，扎实做好公立医院改革和实施国家基本药物制度试点工作；提高县、乡、村医疗机构规范化、标准化、信息化水平；积极发展妇幼保健、疾控、急救和中医药事业；强化甲型H1N1流感、艾滋病、结核病、乙肝等重大传染病防控措施，确保0-15岁儿童国家免疫规划疫苗免费接种全覆盖。认真落实计划生育法规，稳定低生育水平，平衡出生人口性别比。按照国家和省统一部署，切实做好第六次人口普查工作。大力发展文化体育事业。新建一批城乡文化体育设施，规划建设市博物馆等文化场馆；广泛开展群众喜闻乐见的特色文化体育活动；狠抓以网吧监管和“扫黄打非”为重点的文化市场整治，净化文化环境；加强档案、文史工作；全面实现20户以上自然村广播电视“村村通”。

　　加快建设资源节约型和环境友好型社会。认真落实节能减排工作责任制。全面治理和保护城乡环境，突出抓好汉江襄樊段水污染防治，确保县市污水处理厂全部建成投入运行，加快建设市区垃圾发电厂、垃圾转运和污水处理设施，保障汉江水质安全。加强生态环境建设，支持保康县争创国家级生态示范区，力争每个县市区创建1个省级环境优美乡镇、3个省级生态村，努力建成国家环保模范城市。强化节能措施，争取利用国债资金、国家和省节能专项资金，实施高效照明、太阳能利用、建筑节能等一批节能项目，抓好33家高耗能企业节能工程；支持企业实施清洁发展项目，办好9个国家和省循环经济试点。

　　全力维护社会安定和谐。切实加强信访工作，及时处理各种信访问题，努力满足群众的合理诉求；狠抓社会治安综合治理，严厉打击各种刑事犯罪，坚决铲除黑恶势力，全力维护社会安定。支持国防建设，加强“双拥”工作，促进军政、军民团结。巩固和发展民族团结、宗教和顺的良好局面。加强应急能力建设，提高应急管理水平，扎实做好地震、地质、气象等自然灾害防灾减灾工作。严格实施安全生产“一票否决”和责任追究制度，依法查处各类生产安全事故，深化行业安全专项整治，强化安全生产基层基础工作，确保各类安全生产事故指标控制在省政府下达的指标以内。认真执行食品安全法规，深入开展食品安全整顿工作，下大力解决群众反映强烈的食品安全问题。加强农村药品供应和监督网络建设，严格药品质量监管，确保人民群众生命安全。在做好以上各项工作的同时，市政府将集中力量办好关系民生的十件实事。

　　1.启动太平店和隆中污水处理厂建设，建成余家湖污水处理厂，完成南渠和大李沟截污干管工程，整治疏导汉江襄樊段排污口，确保汉江襄樊段水质稳定在Ⅲ类以上。

　　2.新建廉租房7570套，提前一年完成三年建设任务；向人均住房建筑面积13平方米以下的低收入家庭发放住房租赁补贴，缓解低收入家庭住房困难。

　　3.再解决50万人饮水不安全问题，新建沼气用户3.5万个，建设通村水泥(沥青)路700公里，改善农村生产生活条件。

　　4.扩大城乡就业，培训城乡劳动力5.5万人，新增转移农村劳动力8.5万人，新增城镇就业8.6万人，开发公益性岗位安置困难人员3000人。

　　5.增加城乡文化体育设施，新建乡镇综合文化站20个，城市社区流动图书站 20个，农家书屋500家，城乡体育设施90个；建设街心游园3个，免费开放市区公园。

　　6.加强市区集贸市场建设管理，建设规范化集贸市场15个，取缔马路市场30个。

　　7.加大社会救助力度，资助贫困学生15万人以上，将符合条件的农村困难居民全部纳入低保救助范围，为困难群众办理法律援助案件 2300件以上，做好677名0-6岁残疾儿童抢救性康复工作。

　　8.继续抓好市区排水管网改造，再解决6个片区积水问题。

　　9.加快解决国有企业改革遗留问题，完成1万户国有改制、破产企业职工家庭水电改造。

　　10.提高城镇居民医保和新型农村合作医疗补助标准。

　　三、关于政府自身建设

　　做好今年的政府工作，必须深入学习实践科学发展观，切实加强政府自身建设，着力营造促进发展的良好环境。

　　强化服务意识，深入推进“阳光新政”。以建设服务型政府为目标，按照省委、省政府统一部署，积极推进政府机构改革，四月底前完成市政府机构“三定”工作；同时，加快推进政府职能向创造良好发展环境、提供优质公共服务、维护社会公平正义转变。围绕实现在全省“审批项目最少、流程最优、时限最短、收费最少”的目标，推进“阳光新政”制度化、规范化建设。进一步深化行政审批制度改革，继续精简审批事项，大力推行电子政务；完善行政审批电子监察系统，加强行政效能监察。加快建设运转高效的行政服务体系，增强市县行政服务中心的服务功能，启动建设市行政服务中心，实现乡镇便民服务中心和村级便民服务室全覆盖。着眼于营造良好的人文环境，加强公务员和窗口行业服务人员的职业道德教育，力争窗口行业风气社会满意度达到85%以上。建立科学的发展环境考核评价及约束机制，促进发展环境持续改善。

　　强化能力席位意识，提高政府执行力。继续加强政府执行力和能力建设，强化公务员能力席位意识，加快建设一支崇尚学习、善谋实干、勇于创新、勤政为民的公务员队伍。建立有力的执行机制，把各级各部门主要精力凝聚到抓落实、促发展上来。围绕政府工作重点，建立“五个一”推进机制，实行一个项目、一名领导、一套班子、一个方案、一抓到底。建立“四定四保”责任机制，实行定目标、定进度、定措施、定责任，保落地、保开工、保建成、保投产。完善政府目标考核机制，加大对重点项目建设、招商引资进展和重点工作进度的考核力度，强化市政府目标考核和全省县域经济考核优胜单位奖励措施，促进全市形成比发展、争上游的新局面；设立“项目建设突出贡献奖”，激励各级干部在搞服务、抓项目、促发展中建功立业。

　　强化民主法治意识，提高依法行政水平。切实加强民主法制建设。坚持以民主决策促进科学决策。认真执行重大事项专家论证、公开听证制度，主动听取民主党派、工商联、无党派人士和专家学者对政府工作的意见、建议，集中全市人民的智慧促进科学发展。认真执行市人民代表大会及其常委会各项决议，主动加强与人民政协的协商，自觉接受市人大的法律监督和市政协民主监督，认真办理人大代表议案、建议和政协委员提案。加强城市社区和村民自治，新建一批村务公开、民主管理示范村(居)委会。深入开展“五五”普法。强化依法行政监督考核，全面规范行政处罚自由裁量权，认真办理行政复议案件，严格落实执法责任追究制，规范行政执法行为。

　　强化廉洁从政意识，规范行政权力运行。坚决落实党风廉政建设责任制，切实加强对政府工作人员的教育和管理，增强各级公务人员廉洁从政的自觉性。狠抓源头治腐，完善招投标集中管理体制，建立招投标市场综合监管体系，规范招投标秩序；加强经营性土地出让、探采矿权出让以及产权交易、政府采购过程监管，严防腐败滋生；完善国库集中收付制度，加大审计监督力度，严格财政资金运行管理；强化增收节支，建设节约型政府。坚定不移地支持纪检监察机关查处政府系统的腐败案件，树立清正廉明的好风气。

　　今年是执行“十一五”规划的最后一年，我们将在努力完成规划目标任务的同时，精心编制“十二五”经济社会发展规划，使之成为指导今后五年全市经济社会跨越式发展的科学蓝图。

　　各位代表，当前我市正处于集聚跨越式发展能量的关键时期，市政府肩负的任务光荣而艰巨。我们将紧紧依靠省委、省政府和市委的正确领导，按照这次会议的总体部署，团结和带领全市人民，解放思想，抢抓机遇，扎实苦干，为全市生产总值四年翻番打牢基础，为建设更加开放繁荣、更加文明和谐的新襄樊而努力奋斗！
